# Neuromorphic energy economics: toward biologically inspired and sustainable power market design

**DOI:** 10.3389/fncom.2025.1597038

**Published:** 2025-05-27

**Authors:** Aoshuang Ye, Dong Xu, Yichao Li, Jiawei Du, Zhiwei Wu, Junjie Tang

**Affiliations:** ^1^State Grid Shanghai Pudong Electric Power Supply Company, Shanghai, China; ^2^School of Information Science & Technology, Gansu Agriculture University, Lanzhou, China

**Keywords:** neuromorphic computing, energy market design, sustainable power systems, artificial intelligence, bio-inspired optimization

## Introduction

**Figure 1:**
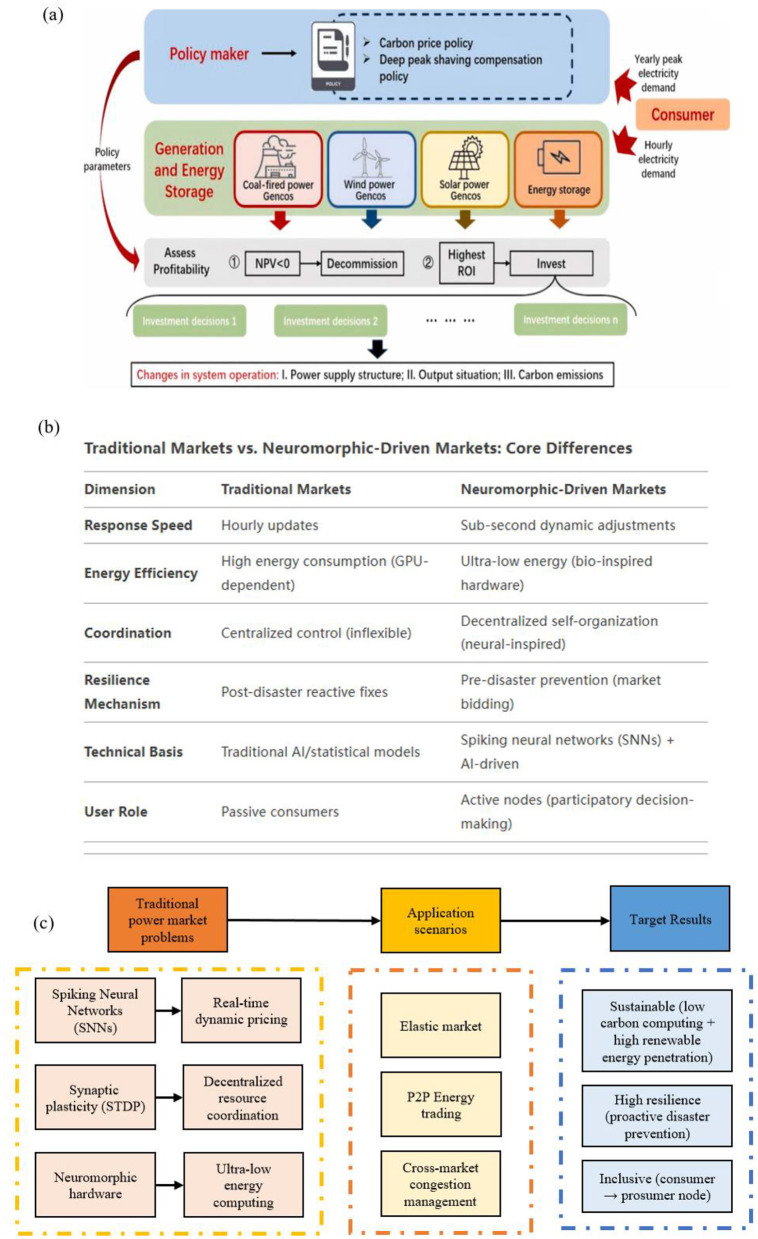
**(a)** Power system structure diagram (Zhao et al., 2025). **(b)** Traditional markets vs. Neuromorphic-driven markets: Core differences. **(c)** Neuromorphic energy economics: Framework overview.

The global energy transition toward renewables has exposed fatal flaws in traditional power market architectures (Grübler and Nakićenović, 1996; Zhao et al., 2025; Khorasany et al., 2018; Xia and Yu, 2025) (Figure 1a). Centralized models, designed for fossil fuel grids (Khan, 2019; Noorollahi et al., 2023; Deshmukh et al., 2021), fail to address renewable (McCauley and Stephens, 2017; Sreedharan et al., 2016) volatility, prosumer participation, and real-time decision demands. The 2021 California blackouts—triggered by solar generation drops and surging EV charging—epitomize this rigidity (Sejnowski et al., 1988; Yuan et al., 2023, 2022a). Concurrently, neuromorphic computing, inspired by the brain's energy-efficient event-driven processing, offers a paradigm shift. Spiking neural networks (SNNs) (Stanojevic et al., 2024; Ayasi et al., 2025) enable microseconds-scale price adjustments with 10–100 × lower energy costs than GPU-based models, while synaptic plasticity (Dampfhoffer, 2023; Peng et al., 2024; Siddique, 2023) rules (e.g., STDP) inspire decentralized DER coordination akin to neural self-organization.

However, translating neuroscience principles into energy economics (Devaraj et al., 2021) requires more than metaphorical borrowing. Neuromorphic hardware (Donati and Valle, 2024) struggles with heterogeneous data (e.g., weather forecasts), and direct mappings of plasticity to incentives risk fairness violations.

This opinion argues for adaptive bio-inspired design—integrating SNNs (Lagani et al., 2023) with hybrid deep learning (LeCun et al., 2015)—to balance efficiency, equity, and interpretability. Evidence includes neuromorphic models outperforming conventional methods in load forecasting, and behavioral economics revealing human energy decisions' collective optimization tendencies. By reimagining grids as brain-like adaptive networks, we propose a roadmap for markets capable of thriving in a high-renewables era.

## Analysis

### Structural rigidity in traditional market design

Contemporary electricity markets (Dagoumas, 2021; Xia et al., 2025) remain anchored in legacy frameworks optimized for centralized, fossil fuel-based systems. These models prioritize stability over adaptability, relying on day-ahead auctions and hourly pricing mechanisms ill-suited to manage the real-time volatility of renewable generation. For instance, solar and wind (Sinha and Chandel, 2015) output fluctuations—driven by weather variability—often lead to supply-demand mismatches, forcing grid operators to deploy costly ancillary services or impose rolling blackouts. Meanwhile, distributed energy resources (DERs) (Fu et al., 2021; Caballero-Pena et al., 2022), such as rooftop solar panels and electric vehicle fleets, are frequently marginalized by markets designed for unidirectional power flows from large utilities to passive consumers. This mismatch stifles innovation, discourages prosumer participation, and perpetuates reliance on fossil fuel backups during renewable lulls.

### Renewable integration and computational bottlenecks

The rapid proliferation of renewables has exposed critical gaps in market scalability and computational efficiency. Existing tools for grid optimization—such as machine learning-driven demand forecasting and congestion management—demand immense computational resources, often offsetting the environmental benefits of clean energy with their carbon-intensive operation (Figure 1b). Additionally, decentralized coordination mechanisms (e.g., peer-to-peer trading platforms) face scalability limits, struggling to balance millions of DERs in real time without sacrificing transparency or fairness. The growing frequency of climate-driven disruptions further strains these systems, as traditional markets lack mechanisms to value resilience or incentivize preemptive investments in grid hardening.

In essence, today's electricity markets operate on outdated logic (Warneryd, 2022; Xia et al., 2018), unable to reconcile the dynamic needs of a renewable-dominated grid with the computational and structural constraints of 20th-century design principles. This dissonance underscores the urgency for paradigm-shifting innovations that prioritize adaptability, efficiency, and equitable participation.

### Neuromorphic computing-driven adaptive power market design

Neuromorphic computing (Marković et al., 2020) introduces event-driven processing to power markets, enabling microseconds-scale responses to renewable volatility. A high-level overview of this neuromorphic energy economics framework is presented in Figure 1c. Unlike traditional models constrained by hourly pricing intervals, spiking neural networks (SNNs) mimic the brain's ability to process temporal data through sparse, energy-efficient communication. For example, during sudden solar generation drops or EV charging surges, SNNs dynamically adjust prices and grid operations in real time, preventing overloads and minimizing reliance on fossil fuel backups. This biological efficiency not only reduces computational energy costs by orders of magnitude but also aligns market behavior with the unpredictable rhythms of renewable energy flows.

By emulating neural networks' decentralized learning, neuromorphic systems empower distributed energy resources (DERs) to autonomously optimize grid stability. Synaptic plasticity principles—such as rewarding prosumers who inject stored solar energy during peak demand—create adaptive incentive mechanisms. These rules enable peer-to-peer energy trading networks to self-organize, balancing local autonomy with global grid needs without centralized oversight. In resilience markets, preemptive resource allocation mimics the brain's predictive capabilities, prioritizing critical loads during extreme events. Such systems transform passive consumers into active “neurons” in a self-healing grid, fostering both economic equity and systemic robustness.

In a simulated urban microgrid, SNNs optimize real-time energy pricing and storage incentives. They monitor energy data from IoT sensors, adjusting prices during peak demand and incentivizing energy storage discharge. Integration with legacy SCADA systems is achieved through a middleware layer, ensuring compatibility and seamless adoption.

### Real-time adaptation through biologically inspired algorithms

Neuromorphic computing revolutionizes power markets by embedding the brain's event-driven efficiency into grid operations. Centralized control and static pricing in traditional markets hinder real-time adaptation to renewable energy fluctuations. Spiking neural networks (SNNs), however, process data through sparse, asynchronous pulses—akin to neurons firing only when necessary. This allows markets to dynamically adjust prices and grid responses within microseconds. For instance, during a sudden cloud cover over a solar farm, SNNs (Yuan et al., 2022b) can instantly reroute power from distributed batteries or adjust demand incentives for electric vehicles, preventing voltage drops without human intervention.

The energy efficiency of neuromorphic hardware further amplifies this advantage. Unlike conventional servers that run continuously, neuromorphic chips activate only when processing spikes, slashing computational energy use by over 90%. This aligns with sustainability goals, ensuring that the carbon footprint of market optimization does not negate the benefits of renewable energy. By mimicking biological systems (Bar-Cohen, 2005), these architectures enable markets to “learn” from past events—such as demand spikes during heatwaves—and preemptively allocate resources, transforming reactive grids into proactive, self-optimizing networks.

### Decentralized coordination and self-organizing resilience

Neuromorphic systems decentralize market control by empowering individual prosumers to act as autonomous decision-makers. Inspired by synaptic plasticity, dynamic incentive mechanisms reward behaviors that enhance grid stability. For example, households with solar-battery systems could earn higher compensation for discharging stored energy during peak demand (Saha et al., 2022), similar to how neurons strengthen connections through repeated activation. This creates a self-reinforcing cycle where participants naturally align their actions with grid needs, fostering organic coordination without top-down mandates.

Peer-to-peer (P2P) energy trading platforms built on neuromorphic principles further illustrate this potential. Instead of relying on energy-intensive blockchain protocols, these systems use lightweight SNNs to validate transactions and balance local supply-demand in real time. In a hypothetical urban microgrid, such a platform could enable factories to sell excess wind power to neighboring schools during low-demand hours, while hospitals prioritize reserved energy for emergencies—all governed by adaptive rules that mimic neural network self-organization.

Resilience is another critical dimension. Neuromorphic markets preemptively allocate resources for extreme events, much like the brain anticipates threats. By analyzing historical weather patterns and real-time sensor data, SNNs predict potential grid failures (e.g., hurricanes or cyberattacks) (Allal et al., 2024) and incentivize pre-emptive investments in redundancy. Customers bidding for “resilience credits” could secure priority power access during disasters, while utilities deploy mobile battery units to high-risk zones. This shifts markets from passive cost-minimization to active risk mitigation, embedding adaptability into their core logic.

### Ethical and scalable market evolution

While neuromorphic computing offers transformative potential, its implementation demands ethical foresight. Algorithmic fairness must be prioritized to prevent exclusion of low-income participants lacking advanced DERs (Simons, 2023). Transparent reward structures—such as capping profit margins for high-frequency traders—can balance efficiency with equity. Additionally, interoperability standards are needed to ensure seamless integration of neuromorphic systems across regions, avoiding fragmented “islands” of innovation.

Dynamic pricing by SNNs may disproportionately affect low-income users without smart devices. Tiered pricing and DER subsidies can mitigate this, ensuring affordable energy access. Memristor-based neuromorphic chips offer cost-effective deployment, potentially enabling resource-limited regions to adopt neuromorphic systems.

Looking ahead, hybrid neuromorphic-quantum systems could tackle grand challenges like continent-scale congestion management. Quantum annealing might resolve complex market equilibria, while SNNs handle real-time adjustments, creating a symbiotic framework. By merging biological inspiration with cutting-edge technology, future markets could achieve unprecedented harmony between human needs, environmental limits, and computational sustainability—ushering in an era where grids evolve as organically as the ecosystems they power.

Quantum annealing can optimize complex market equilibria, while SNNs handle real-time adjustments. However, integrating these components presents challenges such as synchronization and thermal management. Advanced communication protocols and cooling technologies are needed to ensure reliable hybrid system operation.

A regulatory framework is essential to balance innovation with consumer protection, data privacy, and market fairness. It should encourage standardization, mandate transparent data policies, and promote inclusive market participation through subsidies and tiered pricing. Drawing parallels with existing initiatives, such as the EU's Digital Twin of the Ocean, can provide valuable insights for developing such frameworks.

## Discussion

While neuromorphic computing offers groundbreaking efficiency, its practical deployment faces significant hardware constraints. Current neuromorphic chips, though optimized for specific tasks like spike-based processing, struggle to integrate with legacy grid infrastructure and heterogeneous data sources. For instance, weather forecasts, consumer behavior analytics, and equipment health data require diverse computational approaches—ranging from numerical simulations to natural language processing—those existing neuromorphic architectures may not seamlessly support. Hybrid systems combining neuromorphic chips with traditional CPUs or quantum co-processors could address this gap. For example, neuromorphic components might handle real-time pricing adjustments, while classical systems manage long-term policy simulations. However, achieving such symbiosis demands standardized communication protocols and modular hardware designs, ensuring interoperability across evolving technological ecosystems.

The shift toward decentralized, prosumer-driven markets raises critical questions about data ownership and cybersecurity. Neuromorphic systems, by enabling peer-to-peer energy trading and real-time grid adjustments, inherently rely on vast streams of granular user data—from household energy consumption patterns to battery charge cycles. Without robust encryption and decentralized data governance frameworks, these systems risk vulnerabilities to cyberattacks or exploitation by monopolistic entities. Inspired by blockchain's distributed ledger principles, neuromorphic markets could adopt “neuro-secure” architectures where data validation is embedded within spiking neural networks themselves. For instance, SNNs might detect anomalous trading behaviors (e.g., price manipulation) through pattern recognition akin to neural fault tolerance, autonomously isolating malicious actors while preserving user privacy.

The success of neuromorphic markets hinges not only on technical feasibility but also on economic plausibility and human acceptance. The neuromorphic paradigm risks exacerbating global energy inequities if adoption is confined to technologically advanced regions. Developing nations, already burdened by outdated grid infrastructure and financing gaps, may lack the resources to implement neuromorphic systems, widening the divide between “smart” and “legacy” energy markets. To prevent this, international collaborations—modeled after open-source software movements—could democratize access to neuromorphic tools. Shared libraries of SNN algorithms for demand forecasting or resilience planning, coupled with low-cost neuromorphic hardware tailored for off-grid solar communities, might empower regions like Sub-Saharan Africa or Southeast Asia to leapfrog traditional market stages. Such efforts would align with global climate justice goals, ensuring that the neuromorphic revolution benefits all, not just the technologically privileged.

## Conclusion

This paper discusses the new paradigm of neuromorphic computing in power systems. Neuromorphic computing offers a visionary pathway to reimagine electricity markets as adaptive, self-organizing networks that mirror the brain's efficiency and resilience. By embedding event-driven processing and synaptic learning principles into market design, this paradigm addresses the critical gaps in real-time renewable integration, decentralized coordination, and sustainable computation. While challenges such as hardware scalability, ethical governance, and global equity persist, interdisciplinary collaboration and hybrid technologies—like neuromorphic-quantum systems—hold the key to unlocking a future where energy markets evolve dynamically with human and planetary needs. To realize this vision, stakeholders must prioritize co-design of algorithms, hardware, and policies, ensuring that the transition to neuromorphic energy economics is not only technologically transformative but also inclusively equitable.
